# Early Presentation of Achalasia to the Otorhinolaryngology Department

**DOI:** 10.7759/cureus.95151

**Published:** 2025-10-22

**Authors:** Trinyanasuntari Munusamy, How Kit Thong, Primuharsa Putra Sabir Husin Athar

**Affiliations:** 1 Otolaryngology - Head and Neck Surgery, Graduate School of Medicine, KPJ Healthcare University, Nilai, MYS; 2 Otolaryngology - Head and Neck Surgery, Hospital Sultan Ismail, Johor Bahru, MYS; 3 Otolaryngology - Head and Neck Surgery, KPJ Healthcare University, Nilai, MYS

**Keywords:** achalasia, dysphagia, esophagus, rat-tail sign, swallowing

## Abstract

Achalasia is a rare esophageal motility disorder characterized by impaired peristalsis and incomplete relaxation of the lower esophageal sphincter (LES), leading to progressive dysphagia, regurgitation, retrosternal pain, and weight loss. Although the disease is usually detected in adults, pediatric presentations are uncommon and often overlooked, resulting in delayed diagnosis and management. The underlying pathophysiology involves degeneration of inhibitory neurons in the myenteric plexus, causing an imbalance between excitatory and inhibitory neurotransmission, which leads to esophageal outflow obstruction. We describe the case of a 13-year-old female who presented with a two-year history of progressive dysphagia to both solids and liquids, frequent regurgitation, and meal-associated retrosternal pain. She reported significant weight loss of 2 kg over two months despite preserved appetite. A barium swallow demonstrated the characteristic “rat-tail” sign at the gastroesophageal junction, while upper gastrointestinal endoscopy and computed tomography confirmed esophageal dilatation and tapering at the LES. With an Eckardt score of 10, indicating moderate-to-severe disease, the patient was referred for surgical management. She subsequently underwent laparoscopic Heller’s cardiomyotomy with Dor’s fundoplication. Postoperatively, she demonstrated marked symptomatic improvement, weight recovery, and rapid return to normal daily activities. This case highlights the importance of considering achalasia in the differential diagnosis of persistent pediatric dysphagia, which may initially present to specialties such as otorhinolaryngology. Early recognition through imaging and high-resolution manometry is critical to avoid complications such as aspiration, malnutrition, or airway obstruction. Multidisciplinary evaluation and timely surgical intervention remain the cornerstone of treatment, offering excellent long-term outcomes in both pediatric and adult populations.

## Introduction

The term achalasia is derived from the Greek word *khalasis*, meaning “not loosening or relaxing.” It describes a rare esophageal motility disorder characterized by the absence of normal peristaltic contractions and impaired relaxation of the lower esophageal sphincter (LES) following swallowing [1-2]. The condition was first documented by Thomas Willis in 1672, who provided one of the earliest descriptions of achalasia cardia [3].

Epidemiologically, achalasia is considered uncommon, with an annual incidence of approximately one per 100,000 and a prevalence of nine to 10 per 100,000 worldwide [1,4-5]. It shows no consistent predilection for sex, race, or geographic distribution [2]. The majority of cases are diagnosed in adults between the ages of 25 and 60; however, pediatric and adolescent presentations, although rare, are increasingly recognized [6-7]. Pediatric cases are often misdiagnosed as gastroesophageal reflux disease (GERD) or feeding difficulties, leading to delayed diagnosis and progression to more severe complications, including malnutrition, aspiration, and growth retardation [6-7].

The exact etiology of achalasia remains unclear. Pathophysiological studies demonstrate degeneration of inhibitory ganglion cells in the myenteric plexus, leading to impaired nitric oxide and vasoactive intestinal peptide (VIP)-mediated LES relaxation [1,8-9]. This imbalance between excitatory cholinergic and inhibitory neurotransmission results in functional obstruction at the gastroesophageal junction. Current evidence suggests that the disease process may also involve autoimmune and inflammatory mechanisms, potentially triggered by viral infections in genetically predisposed individuals [6-8].

Clinically, achalasia presents most frequently with gradually progressive dysphagia to both solids and liquids, regurgitation of undigested food, retrosternal chest pain, and weight loss [1,2]. In some cases, patients may also develop respiratory manifestations, such as chronic cough, aspiration pneumonia, hoarseness, or even airway compromise [7,10]. Because of this broad symptom spectrum and overlap with other gastrointestinal or respiratory disorders, diagnosis is often delayed. While esophageal manometry remains the gold standard diagnostic tool, barium swallow demonstrating the characteristic “rat-tail” or “bird-beak” narrowing, along with endoscopic findings of a dilated esophagus with stasis, are valuable in confirming the diagnosis [1,6,9].

This case report highlights the presentation of achalasia in a 13-year-old female who initially sought care in an otorhinolaryngology department. Her case underscores the importance of multidisciplinary awareness, as early recognition and treatment are critical to prevent long-term morbidity.

## Case presentation

**Figure 1:**
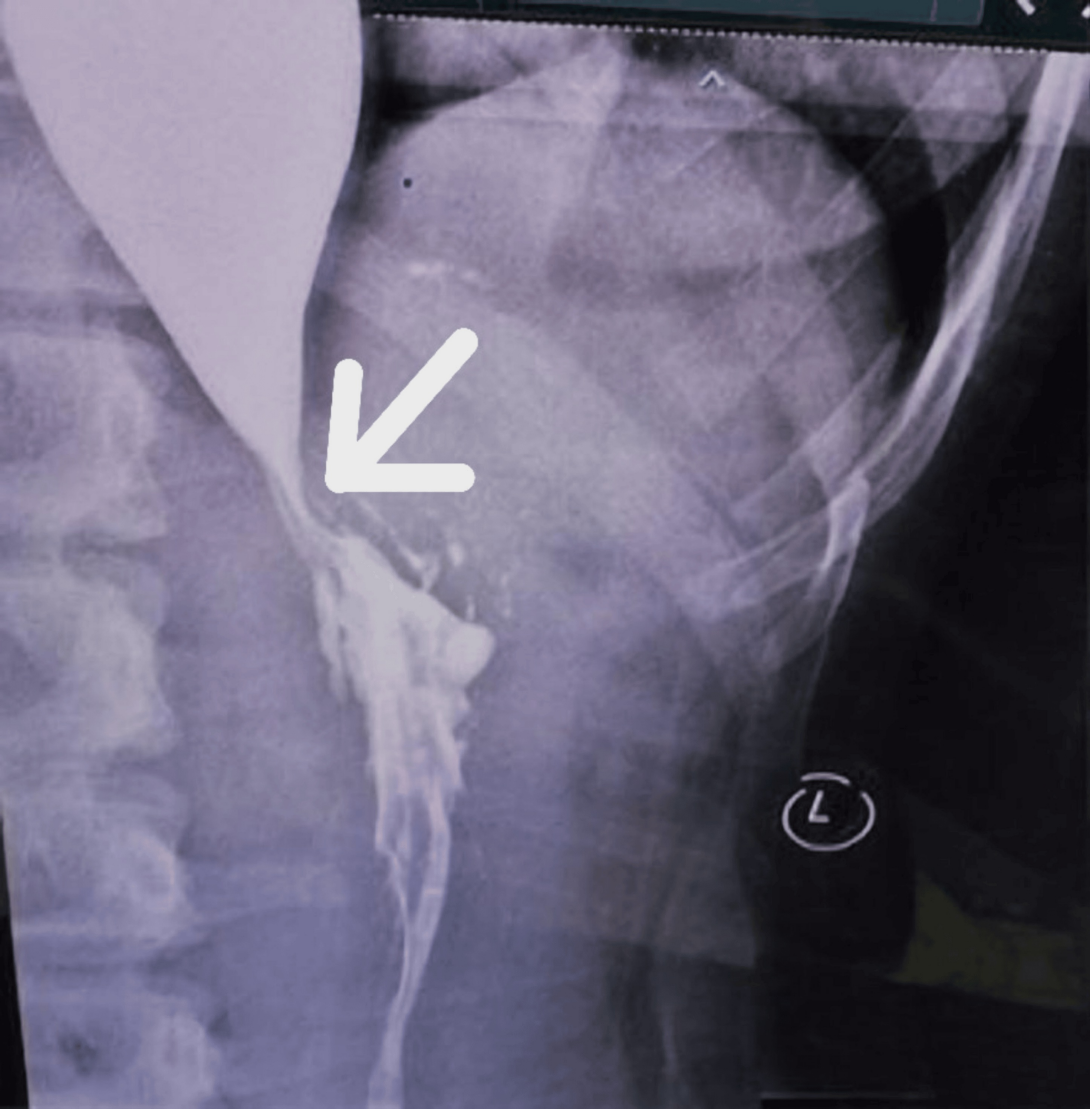
Frontal barium swallow radiograph demonstrates a dilated esophagus with abrupt tapering at the gastroesophageal junction, producing the classical “rat-tail” configuration (arrow). Contrast hold-up is evident proximal to the narrowing. The esophageal wall appears smooth without shouldering or irregularity, making malignancy less likely. Frontal barium swallow showing a dilated esophagus with distal “rat-tail” tapering (arrow), consistent with achalasia cardia. The “L” marker denotes the patient’s left side.

**Figure 2:**
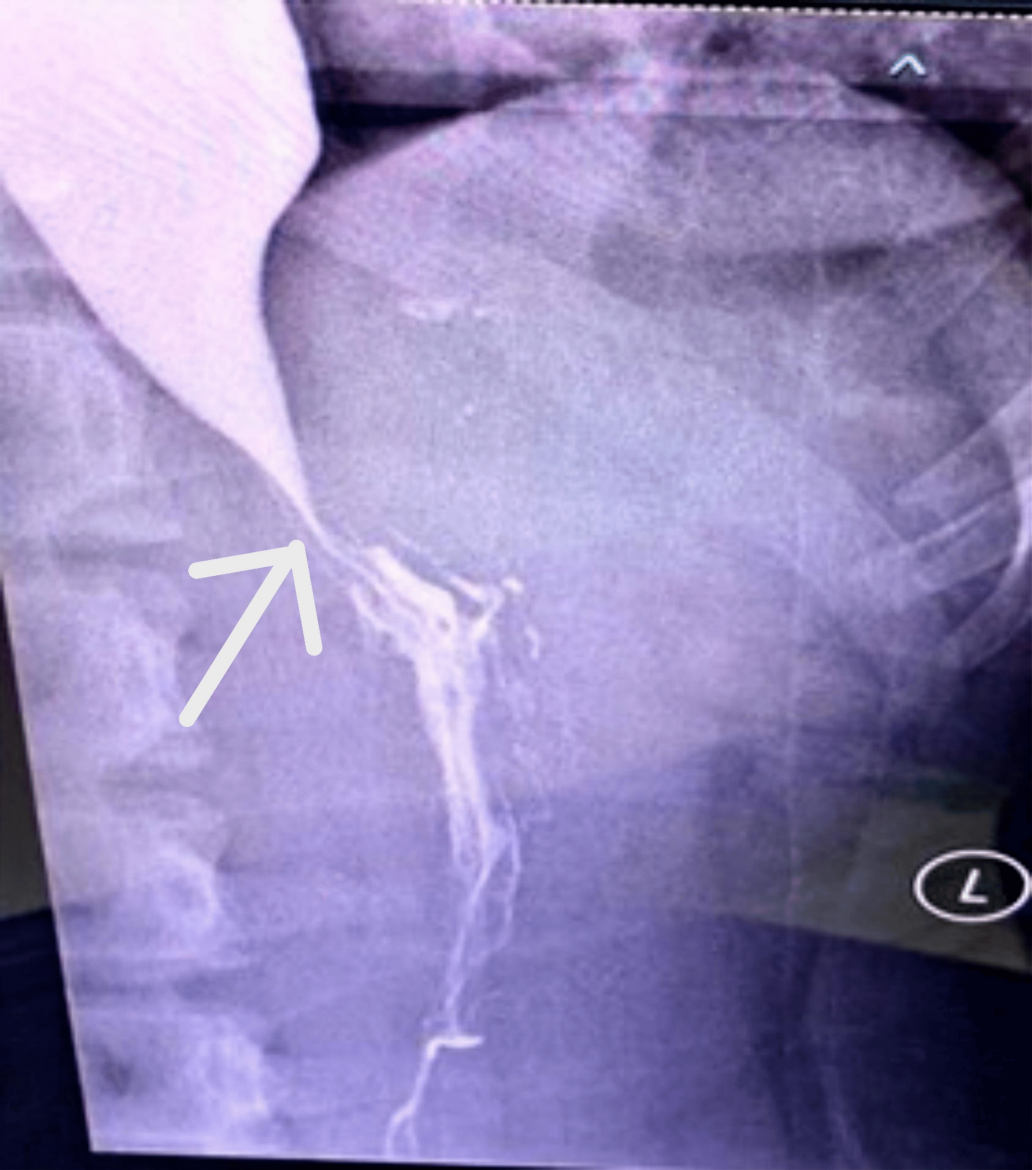
Dilatation of the esophagus Frontal barium swallow demonstrating a dilated esophagus with distal smooth tapering (“rat-tail” sign, arrow), consistent with achalasia cardia. The “L” marker identifies the patient’s left side.

This is a case of classic presentation of achalasia in a 13-year-old female, characterized by progressive dysphagia, regurgitation, retrosternal pain, and unintentional weight loss over the past two years, exacerbated in the last two months. Despite her symptoms, she maintains a good appetite and denies other concerning symptoms like fever, nausea, or bowel changes. Upon examination, no abnormalities were noted, but a barium swallow study revealed dilatation and stasis in the thoracic oesophagus, tapering at the LES, and narrowing at the gastroesophageal junction-classic findings suggestive of achalasia. In our case, diagnostic investigations provided a comprehensive picture confirming the diagnosis of achalasia cardia. The barium swallow revealed the classic "rat tail appearance," indicative of oesophageal narrowing as per Figures 1-2.

**Figure 3:**
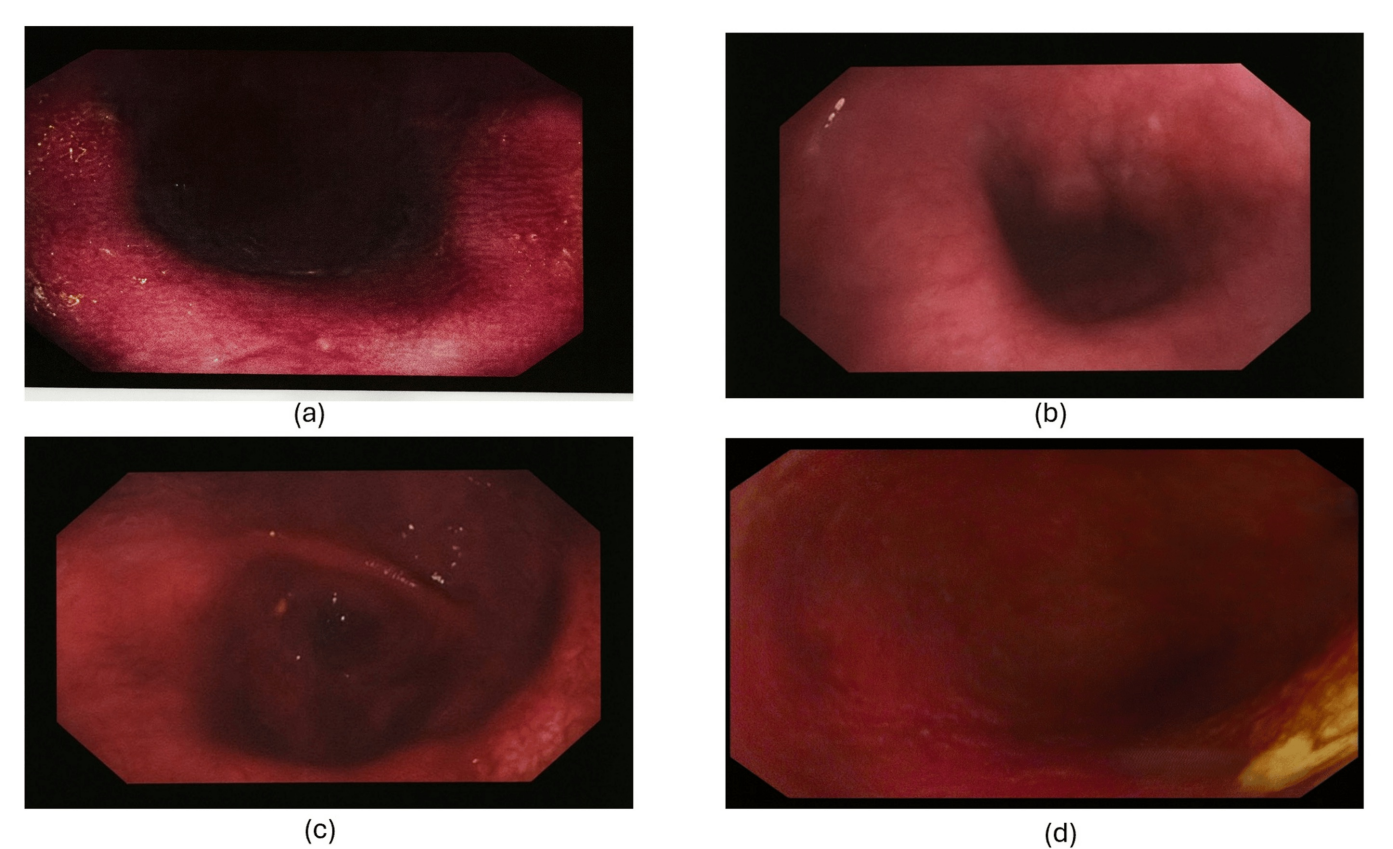
Endoscopy of grossly dilated esophagus devoid of peristalsis Figure 3a-3d show the upper gastrointestinal endoscopy unveiled a grossly dilated esophagus devoid of peristalsis, consistent with achalasia.

Upper gastrointestinal endoscopy unveiled a grossly dilated oesophagus devoid of peristalsis, consistent with achalasia as per Figure 3.

**Figure 4:**
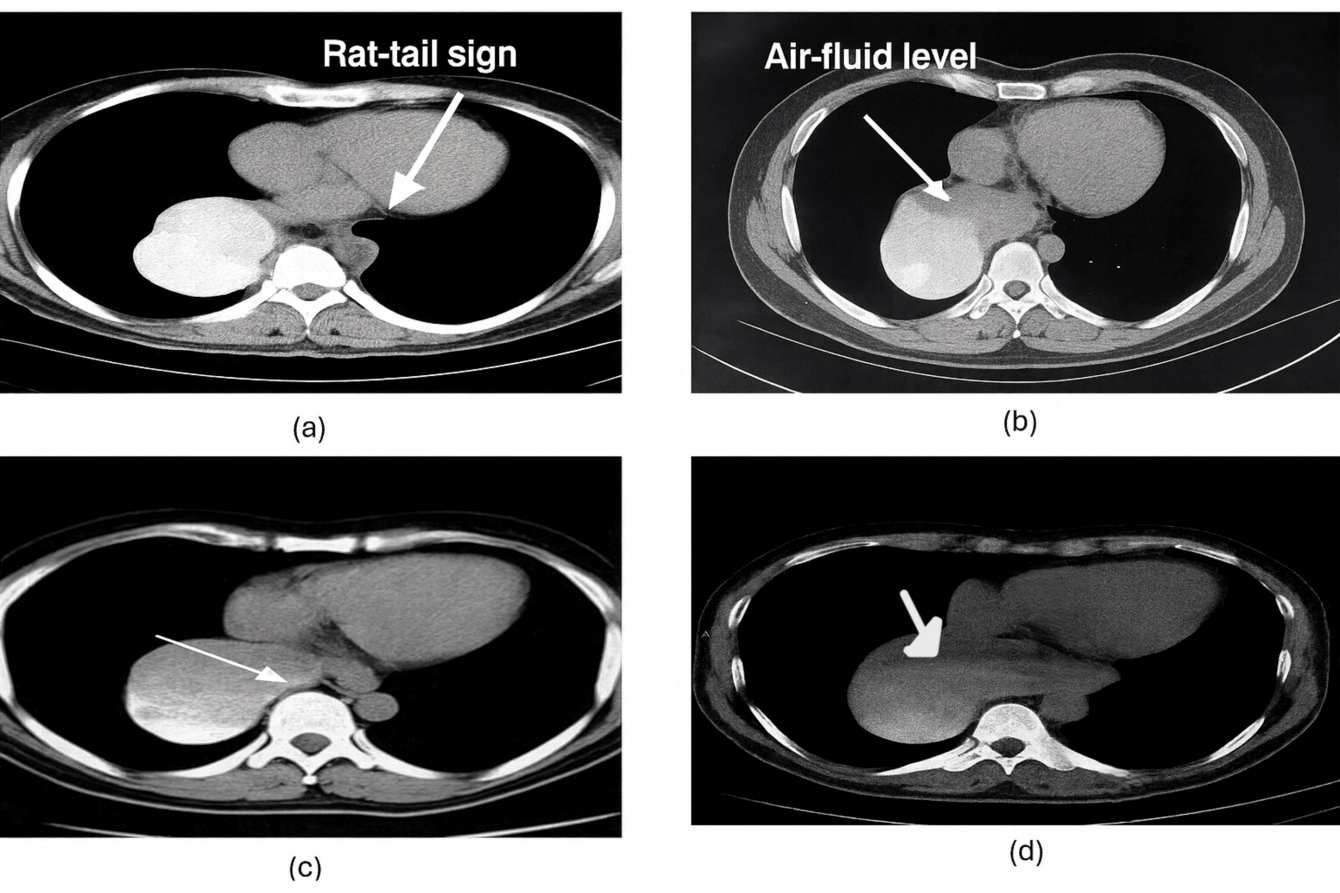
Abdominal CT showing dilatation of the esophagus with rat-tail sign and air fluid level Figure 4a-4d show the axial contrast-enhanced CT images  showing a markedly dilated esophagus with distal smooth tapering at the gastroesophageal junction (arrows). (a): There is tapering of the distal esophagus as it approaches the gastroesophageal junction, forming a conical narrowing resembling a rat’s tail — hence termed the “rat-tail sign.” Proximal to this narrowing, the esophageal lumen appears moderately dilated and fluid-filled, suggesting stasis of ingested material and secretions. The esophageal wall may appear slightly thickened, but without any mass lesion or mucosal irregularity, distinguishing it from a malignancy-related stricture. The surrounding mediastinal structures (heart, descending aorta, and vertebral body) are normal, indicating a functional rather than infiltrative obstruction. This sign is characteristic of achalasia cardia, where failure of the lower esophageal sphincter (LES) to relax results in a smooth, symmetric tapering at the distal esophagus. (b): The esophagus is grossly dilated and fluid-filled, with a distinct horizontal air-fluid interface visible in the lumen, this is the air-fluid level. The air above represents swallowed air trapped due to the LES obstruction, while the dependent fluid layer corresponds to retained saliva and food debris. The air-fluid level indicates stasis and poor emptying of the esophagus, another supportive finding of achalasia or esophageal outflow obstruction. (c): The arrow is pointing toward the distal esophagus just above the diaphragm, which shows a dilated, fluid-filled esophageal segment tapering toward the gastroesophageal junction. This supports the presence of achalasia, continuing the “rat-tail” appearance seen in (a). (d): The arrow indicates the proximal fluid-filled esophagus extending upward from the narrowed gastroesophageal junction. It highlights the esophageal dilatation due to stasis of fluid and air — part of the same achalasia spectrum showing air-fluid level.

Computed tomography further supported the diagnosis, showing a gradual tapering at the esophagogastric junction and gross distension of the esophagus with a thin and smooth wall, accompanied by an air-fluid level as per Figure 4.

With an Eckardt score of 10 (Table 1), indicating moderate to severe symptoms, the patient was promptly referred to an upper gastrointestinal surgeon for further management. This case underscores the importance of recognizing and diagnosing achalasia early, especially in young patients, to initiate appropriate treatment and improve their quality of life.

**Table 1:** Eckhardt score The Eckardt score is given on the above mentioned presentation, i.e., the dysphagia degree with score of 3 happens during each meal, regurgitation of contents with score of 3 during each meal, pain in chest or retrosternal pain with score of 3 during each meal, and weight loss with score of 1 , <5 kg upon presentation.

Score	Dysphagia	Regurgitation	Retrosternal pain	Weight loss (kg)
0	None	None	None	None
1	Occasional	Occasional	Occasional	<5
2	Daily	Daily	Daily	5 to 10
3	Each meal	Each meal	Each meal	>10

## Discussion

LES pressure and relaxation are controlled by a balance between excitatory neurotransmitters like acetylcholine and substance P, which increase pressure, and inhibitory neurotransmitters like nitric oxide and vasoactive intestinal peptide (VIP), which promote relaxation. This balance ensures proper oesophageal function and prevents LES dysfunction-related disorders [1-3].

Achalasia is marked by the absence of noradrenergic, noncholinergic, and inhibitory ganglion cells in the oesophagus, resulting in an imbalance between excitatory and inhibitory neurotransmission. As a result, the esophageal sphincter becomes hypertensive and unable to relax. It is considered an uncommon disease, with an incidence rate of 10 cases per 100,000 persons and a morbidity rate of one per 100,000 [1,2]. With a male-to-female ratio of 1:1, achalasia is most frequently detected in individuals between the ages of 25 and 60 [1]. In this instance, what makes the case distinctive is the patient's age: she is a 13-year-old female. Despite her young age, she exhibits symptoms consistent with achalasia, illustrating that this condition can manifest in individuals of various age brackets, including adolescents.

Common symptoms include dysphagia, regurgitation, difficulties with swallowing, chest pain, heartburn, and weight loss [1-3]. Respiratory symptoms may encompass coughing, asthma-like features, chronic aspiration, hoarseness, and a sore throat [1-5]. If a patient develops increasing difficulty swallowing solids and liquids, as well as regurgitation of food and saliva, it is important to diagnose them with achalasia. Physical examinations are often not helpful. In this case, the patient experienced dysphagia for a few years, along with difficulties swallowing solid food, retrosternal pain, and weight loss. Physical examination results were normal. In the provided presentation, the Eckardt score assesses the severity of symptoms, including dysphagia, regurgitation, chest pain, and weight loss, serving as a crucial tool for evaluating achalasia’s treatment efficacy [2]. While impaired belching is an uncommon symptom in achalasia, some patients may experience it. In addition, a subset of patients may present with emaciation and ulcers in the oral cavity, often attributed to the regurgitation of acidic food material [2].

The clinical diagnosis of achalasia is typically confirmed using a barium swallow esophagus X-ray, which is considered the gold standard diagnostic modality. One of the hallmark features observed in this imaging study is the “rat-tail appearance,” characterized by a tapered narrowing of the esophagus resembling a sharp wooden pencil tip [1-3,7]. Endoscopic examination is also valuable in assessing patients with achalasia, although it may not always reveal specific abnormalities. Another important diagnostic tool is oesophageal manometry, which demonstrates an elevated pressure at the gastroesophageal (GE) junction, typically double the normal pressure (40 mmHg), and a lack of relaxation tone in this region [6].

Esophageal manometry demonstrated a complete absence of peristalsis and provided quantitative data supporting the diagnosis. Specifically, the highest distal contractile integral (DCI) was 71, the mean DCI was 13 mmHg/s, the lower oesophageal sphincter pressure was elevated at 46 mmHg, and the integrated relaxation pressure (IRP) was notably high at 38 mmHg, consistent with type I achalasia based on a study on children [6].

The management of achalasia primarily aims to alleviate symptoms by reducing the pressure at the LES [2]. Once the obstruction is addressed and relieved, food can pass through the oesophageal body via gravity, aided by any remaining peristaltic activity. Endoscopic treatment for achalasia typically involves injecting botulinum toxin directly into the lower oesophageal sphincter (LES) [1,9]. This action inhibits the release of acetylcholine, restoring the balance between excitatory and inhibitory neurotransmitters. However, while this approach can provide temporary relief, its efficacy is limited. Studies have shown that only around 30% of patients who undergo endoscopic botulinum toxin injection experience sustained relief from dysphagia one year after treatment [1]. Furthermore, most patients require repeated injections over time to maintain symptom control.

In addition, calcium channel blockers and nitrates are sometimes prescribed to manage achalasia by reducing the pressure of the LES [1,2,4,9]. While this approach offers relief for approximately 10% of patients, it can serve as a valuable option, especially for individuals who are not suitable candidates for surgical interventions [2]. Another treatment modality includes pneumatic dilation, often recommended as an alternative treatment option for achalasia [1,9]. However, many surgeons regard laparoscopic Heller myotomy as the optimal primary modality for managing patients with achalasia cardia. This minimally invasive procedure involves cutting the muscles of the lower oesophageal sphincter to facilitate easier passage of food into the stomach [1,2,9]. While Heller myotomy is highly effective in relieving symptoms of achalasia, there is a potential downside. When combined with a partial fundoplication performed thoracoscopically, there is a notable incidence of gastroesophageal reflux disease (GERD) post-surgery [2].

The patient was promptly referred to an upper gastrointestinal surgeon, where she underwent laparoscopic Heller cardiomyotomy and Dor’s fundoplication. Following the procedure, significant clinical improvement was observed. Subsequent fortnightly follow-ups revealed continued progress, and the patient was able to resume her normal diet. As her recovery advanced, follow-up appointments were scheduled monthly in the outpatient department. Remarkably, after just one month, the patient was able to return to her school activities, signalling a successful outcome of the surgical intervention and a significant improvement in her quality of life.

## Conclusions

We present the case of a 13-year-old female adolescent diagnosed with achalasia cardia. Her initial presentation to the ear, nose, and throat (ENT) outpatient department, coupled with diagnostic assessments confirming her condition, facilitated prompt referral to an upper gastrointestinal surgeon for specialized care. Following appropriate treatment, the patient experienced significant clinical improvement, highlighting the importance of early diagnosis and interdisciplinary collaboration in managing complex medical conditions such as achalasia
